# The First Essential to Successful Co-Operation

**Published:** 1914-03-21

**Authors:** 


### THE FIRST ESSENTIAL TO SUCCESSFUL CO-OPERATION.

The association of The Hospital with associations of members of the medical profession, and especially with the non-panel practitioners and those connected with the public services and with hospitals and Poor-Law institutions, which is continuously growing closer, has probably given rise to inquiries as to how far we could help the doctor's wife to secure by co-operation the best markets, backed by a practical knowledge of the earning power of money which would help her economically in many ways. The article by "A Doctor's Wife" which we publish on the first page of this Supplement is fruitful in suggestion and, being from the pen of one who is herself a qualified member of the profession, possesses a force and directness of great value. Its writer is not now in practice, because she is devoted to the work and companionship of her husband, who is an eminent consultant in large practice. Her article is therefore one which should carry weight and conviction to doctors' wives all over the kingdom. It has made a deep impression upon ourselves, and we have given some months to the consideration of the proposals and scheme to which the writer refers.

We find that, providing doctors' wives are prepared to co-operate in sufficient numbers and to join the Association referred to, it should be practicable to organise it in such a way as to increase the earning power of their available income now expended upon the home and domestic matters by an average of from 10 to 20 per cent. *Food in any form the Association cannot deal with.* This would mean in practice an addition to those portions of the income spent *otherwise than on foodstuffs* to the extent of one-fifth to one-tenth of such income. We merely mention this as a sort of key to the possibilities which the doctor's wife should procure through co-operation, providing the idea appeals at the outset to a sufficient number of doctors' wives to enable the scheme of our correspondent to be brought into practical operation without delay. Everything else is in an advanced state to proceed.

**To enable the plan to be thoroughly tried it is desirable that from 1,000 to 5,000 doctors' wives should sign and return the coupon which will be found on page 678 of the present issue of "The Hospital."**

**We are enabled to give four weeks to the doctors' wives to consider these proposals, and if One Thousand send in the signed coupon and intimate their willingness to try the scheme for one year, we propose in "The Hospital" on May 2 to go forward with the Association. The first thousand wives whose coupons are received as joining the Association will have precedence, for with one thousand we can go forward energetically with the co-operation of doctors' wives.**

We know by the experience we have gained already, apart from any economical saving, that it is possible to render substantial service through several of the departments which it is hoped to include in the work of the Association of Doctors' Wives. Some of these ways will be readily understood from the short notes under various headings which follow this article.

